# Off-Pump Triple Coronary Artery Bypass Grafting in a Patient with Situs Inversus Totalis: Case Presentation and a Brief Review of the Brazilian and the International Experiences

**DOI:** 10.5935/1678-9741.20160021

**Published:** 2016

**Authors:** Carlos Junior Toshiyuki Karigyo, Felipe Batalini, Alexandre Noboru Murakami, Rogério Toshio Teruya, Francisco Gregori Júnior

**Affiliations:** 1 Department of Cardiac Surgery at Hospital Regional João de Freitas, Arapongas, PR, Brazil.; 2 Department of Internal Medicine at Boston University Medical Center, Boston, United States.; 3 School of Medicine at State University of Londrina, Londrina, PR, Brazil.

**Keywords:** Coronary Artery Bypass, Off-Pump, *Situs Inversus*, Dextrocardia

## Abstract

A 76-year-old man with *situs inversus totalis* underwent a successful off-pump three-vessel coronary artery bypass surgery. The postoperative course was uneventful, and the patient was discharged 8 days later. At 9-month follow-up a coronary computed tomography angiography confirmed the viability of all of the grafts, and one year after the operation the patient remained asymptomatic. It comprises the fifth Brazilian case of a coronary surgery in a patient with *situs inversus totalis* and the first one of the country of a coronary artery bypass surgery without the use of the cardiopulmonary bypass in this condition.

**Table:** 

Abbreviations, acronyms & symbols	
BIMA	= Bilateral mammary arteries
CAD	= Coronary artery disease
CPB	= Cardiopulmonary bypass
CT	= Computed tomography
LCA	= Left main coronary artery
LIMA	= Left internal mammary artery
PDA	= Posterior descending artery
RIMA	= Right internal mammary artery

## INTRODUCTION

The *situs inversus* condition can be associated to levocardia or dextrocardia. In levocardia the apex of the heart is situated on the left side of the body, and in dextrocardia the apex of the heart is on the right side. *Situs inversus* associated to dextrocardia is also termed as *situs inversus totalis*, with cardiac apex and atrial chambers as well as abdominal organs being a mirror image of the normal anatomy. *Situs inversus totalis* is a rare condition with an incidence of 1:10.000 in the population. Individuals with this condition commonly survive long enough to develop atherosclerotic coronary artery disease (CAD), with an incidence similar to the general population, and then may also undergo interventional or surgical procedures when indicated^[1]^. There are very few reports about coronary artery bypass surgery in patients with this condition in the world, and less than a half dozen cases in Brazil^[1-5]^. As far as we know, this is the first report of an off-pump coronary artery bypass grafting in a patient with *situs inversus totalis* in Brazil.

## CASE

This study was performed after written informed consent from the patient and approval by the Ethics Committee of the João de Freitas Regional Hospital (Arapongas, Paraná, Brazil).

**Fig. 1:**
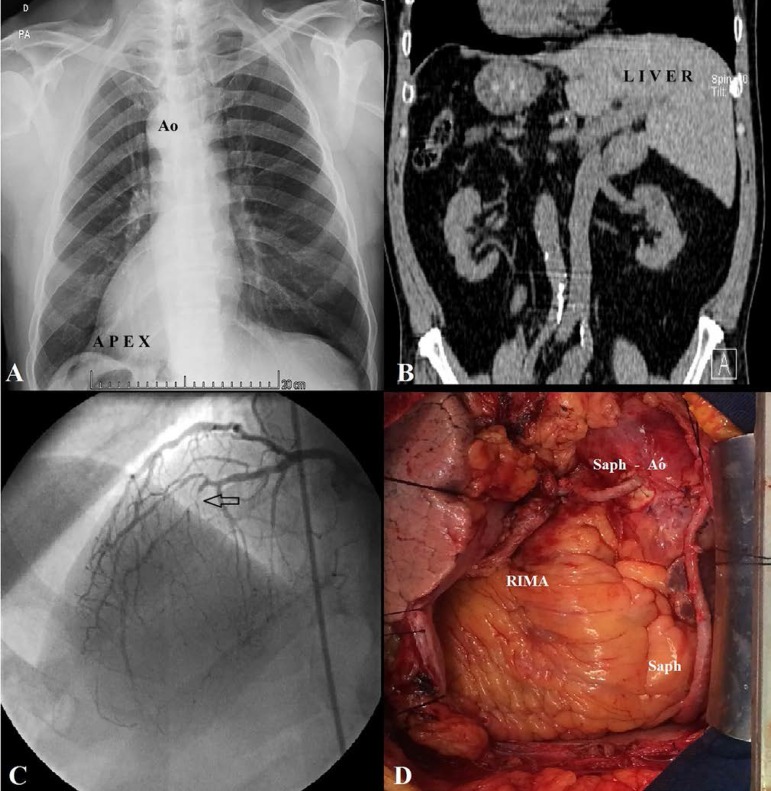
(A) Preoperative chest roentgenogram showing dextrocardia. (B) Abdominal CT demonstrating situs inversus totalis. (C) Coronary angiography. (D) Intraoperative view after complete revascularization.

A 76-year-old man (73 kg and 169 cm) with a diagnosis of *situs inversus totalis* - that was found during the military service recruitment - and hypertension was admitted with a three month story of chest pain associated to dyspnea on moderate efforts despite optimized pharmacological therapy. The chest roentgenogram was normal except for dextrocardia and an abdominal computed tomography (CT) confirmed a *situs inversus totalis* (Figures 1A and 1B). He underwent a coronary angiography, which revealed a triple vessel coronary artery disease (Figure 1C). The patient was then referred to surgical treatment.

### The Surgical Technique

After induction of general anesthesia, the procedure was initiated through a median sternotomy, with the surgeon standing on the right side of the patient to open the chest. Besides presenting with a good contractile performance, we observed that the heart presented with an exact mirror image of a normally positioned heart: the apex of the heart on the right, the right atrium on the left, the pulmonary artery on the right side of the aorta and the left ventricle on the right. Then after, with the surgeon standing on the left side of the patient, the right internal mammary artery (RIMA) was harvested in the standard manner as pedicle. The pericardium was then opened and deep pericardial traction sutures were placed to adequately expose both lateral and posterior walls of the heart. With these technical arrangements the visualization of the coronary arteries was feasible enough to perform the anastomosis without using mechanical stabilizers - careful manual stabilization plus transient and minimal inotropic support assured hemodynamic stability during the operation in this case. Three distal anastomoses were performed, the RIMA was anastomosed to the left anterior descending artery (LAD) and the two saphenous vein grafts to the first obtuse marginal branch and to the posterior descending artery (PDA, Figure 1D). The surgical procedure was performed without the use of cardiopulmonary bypass (CPB). Except for the mirror-image anatomy, the surgical technique had not been widely modified compared to a patient with *situs solitus.*

### The Postoperative Course

**Fig. 2:**
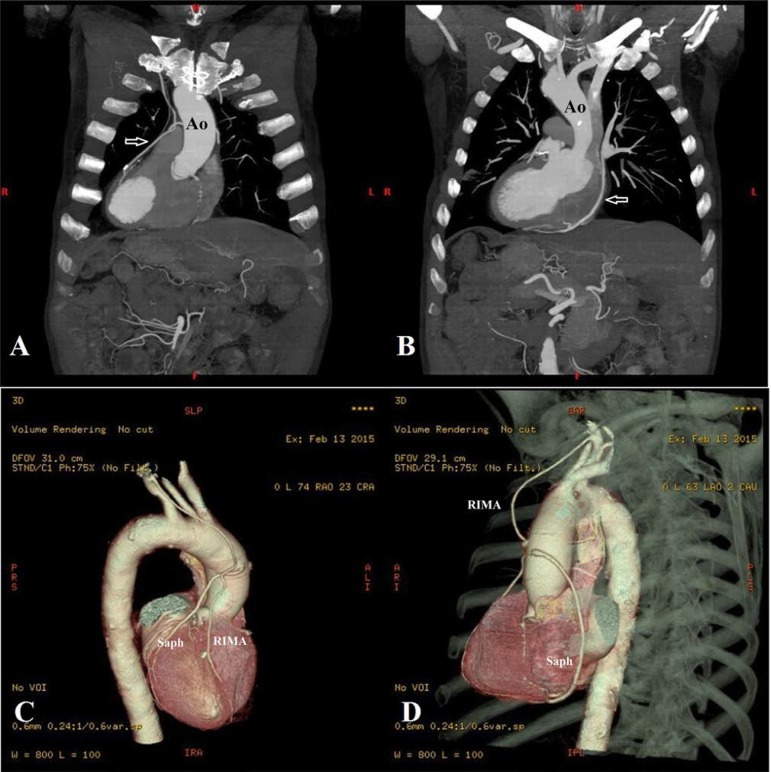
Postoperative CT angiography indicating (A) RIMA anastomosed to the LAD artery (arrow); (B) Saphenous vein grafted to the PDA (arrow); (C) three dimensional front view and (D) lateral view.

The patient's postoperative course was uneventful, he remained stable without the need for inotropic support and was weaned from mechanical ventilation after a few hours of his arrival at the cardiac intensive care unit. There were no surgical and medical complications during his time in hospital and he was discharged home 8 days later. At a 9-month follow-up the patient had undergone a CT coronary angiography that confirmed the viability of all of the grafts (Figure 2). At a one-year follow-up the patient remained in good clinical condition and with no further complications.

## DISCUSSION

Dextrocardia with *situs inversus totalis* is a rare congenital abnormality characterized by the development of visceral organs on the opposite side of its topography, like a mirror image. Hieronymus Fabricius (1537 - 1619) was an Italian anatomist and surgeon who first described *situs inversus* in 1606^[1,4]^. The combination of a rare condition such as dextrocardia and *situs inversus totalis* with a cardiac disease has been reported sporadically in medical literature, generally related to congenital anomalies, and recent reports regarding to atherosclerotic CAD has emerged. Patients with this condition are so vulnerable to be affected by CAD than general population, but the few number of only recently reported cases may be resulted from advances in life expectancy of the population in combination with medical advances in diagnostic and therapeutic fields, and more importantly, with the rarity of this condition by itself^[1]^.

The first cases of coronary artery bypass surgery in patients with *situs inversus* were reported by Grey and Cooley, in 1981, and by Irving et al., in 1982^[1,5]^. Previously, Richardson et al., in 1974, described a case of a ventricular aneurysm resection after myocardial infarction in a patient with persistent ventricular tachycardia ^[1]^.

### The Brazilian Experience

In 1988, Abensur et al.^[1]^ published the first report of coronary artery bypass grafting using the RIMA in *situs inversus totalis*, and this case marked the first Brazilian report of coronary bypass surgery in this kind of patient. Only 14 years later, Rosa et al.^[2]^, in 2002, reported the second case in the country, performing a triple-vessel revascularization with CPB utilizing RIMA and two saphenous vein grafts. Pego-Fernandes et al.^[3]^ reported a case in which they performed an on-pump quintuple coronary artery bypass grafting and Saadi et al.^[4]^ reported another case in which they performed a triple on-pump coronary bypass grafting. In these three operations, the only change in the surgical setup was the surgeon standing in the left side of the patient, not altering the rest of the scenario. On the other hand, the surgical setup in these situations may be completely modified when the complexity requires. The Brazilian experience is summarized in Table 1.

**Table 1:** The Brazilian experience with coronary artery bypass grafting in a patient with *situs inversus totalis.*

Authors	Year	Age (years)	Gender	Disease	Distal anastomosis	Grafts	Pump	Surgeon side
Karigyo et al.	present	76	M	3VD	3	RIMA, Saphenous vein	Off	Left
Saadi et al.^[4]^	2007	78	F	3VD*	3	RIMA, Saphenous vein	On	Left
Pêgo-Fernandes et al.^[3]^	2007	63	M	5VD	5	RIMA, Saphenous vein	On	Left
Rosa et al.^[2]^	2002	53	F	1VD (LCA)	3	RIMA, Saphenous vein	On	Left
Abensur et al.^[1]^	1988	38	M	1VD	1	RIMA	On	Not mentioned

F=female; LCA=left main coronary artery; M=male; RIMA=right internal mammary artery; VD=vessel disease

* Presence of aneurysms in the left descending coronary artery

### International Experience (Off-Pump Procedures)

The first cases of Grey and Cooley were reported in 1981, but using the CPB circuit. Twenty years later, Tabry et al.^[6]^ were the first to publish a case of a coronary artery bypass surgery with the off-pump technique, in which both mammary arteries (the free LIMA anatomosed to the *in situ* RIMA) and a saphenous vein graft were sequentially anastomosed. After that report, a few number of authors have published similar cases, with some differences in their technical options. These data are presented in Table 2.

**Table 2:** The international experience with coronary artery bypass grafting in a patient with *situs inversus totalis.*

Authors	Year	Country	Age (years)	Gender	Disease	Distal anastomosis	Grafts	Surgeon side
Tabry et al.^[6]^	2001	USA	42	M	3VD	4	BIMA, Saphenous vein	Left
Stamou et al.^[7]^	2003	USA	65	F	2VD	2	RIMA, Saphenous vein	Both
Bonde & Campalani^[8]^*	2003	UK	82	M	2VD	2	RIMA, Saphenous vein	Left
Bonanomi et al.^[9]^	2004	USA	72	F	2VD	2	RIMA, Saphenous vein	Not mentioned
Abdullah & Mazalan ^[10]^	2004	Malaysia	56	M	3VD	3	Saphenous vein	Right
Kuwata et al.^[11]^	2004	Japan	49	M	3VD	5	BIMA, Radial artery	Left
Ennker et al.^[12]^	2006	Germany	82	F	unknown	2	RIMA	Left
Chakravarthy et al.^[13]^	2008	India	41	M	3VD	2	LIMA, Radial artery, Saphenous vein	Right
Chakravarthy et al.^[13]^	2008	India	81	M	3VD	3	RIMA, Saphenous vein	Both
Yamashiro et al.^[14]^	2009	Japan	73	M	3VD	3	BIMA, Radial artery	Right
Romano et al.^[15]^‡	2010	Italy, Venezuela	59	F	1VD	1	RIMA	Right
Arrigoni et al.^[16]^	2010	The Netherlands	67	M	3VD	3	RIMA, Radial artery	Left
Takahashi et al.^[17]^	2011	Japan	83	M	3VD	3	BIMA, Saphenous vein	Right
Mouline & Vallely ^[18]^	2013	Australia	81	M	1VD	2	BIMA	Not mentioned
Su et al.^[19]^‡	2013	China	65	F	3VD	3	RIMA, Saphenous vein	Both

BIMA=bilateral mammary arteries; F=female; LCA=left main coronary artery; M=male; RIMA=right internal mammary artery; VD=vessel disease

* Conversion to on-pump technique due to hemodynamic instability

‡ Minimally invasive technique

### Minimally Invasive Approach

Although not widely diffused in our country, minimally invasive techniques can be employed in these cases, like demonstrated only by a couple of authors. The first report was described by Romano et al.^[15]^, in 2010, in which they performed a right anterior small thoracotomy (11 cm in length) to access the chest and mobilize the RIMA in a skeletonized fashion. Distal anastomosis to the LAD artery was achieved by the standard beating-heart technique and the surgeon standing on the right side of the patient. The authors claimed more complete and normal view of the operative field using such technique and arrangement. Su et al.^[19]^, in 2013, described an off-pump triplevessel coronary bypass surgery with a partial sternotomy incision (10 cm in length), in which the surgeon performed the distal anastomosis standing on the left side of the operating table and after chose the right side to perform the proximal anastomosis because of the better exposure of the ascending aorta.

## TECHNICAL AND FINAL CONSIDERATIONS

One of the most important facts in these cases is related to the best position of the surgeon on the operating table specially when considering off-pump procedures, due to the natural challenges during beating-heart operations and the mirrored operative field in the *situs inversus* condition. Besides of our report, we found 15 patients operated without the use of CPB. In 2 of them there was no clear mention about the surgeon side. In 5 patients, the operations were performed with the surgeon standing on the right side of the patient, in another 5 on the left side and in 3 the surgeon stood on both sides. Bonde & Campalani^[8]^ strongly recommended performing the surgery from the left side, but some authors simply felt at ease to perform in this way^[20]^. Chakravarthy et al.^[13]^ reported a series of two cases, in the first standing on the right side, but in the second case they chose to switch the positions, concluding that such cases brought extra technical problems even if standing on the left side. Chakravarthy et al.^[13]^ and Su et al.^[19]^ performed the distal anastomosis standing on the left side and the proximal anastomosis on the right side. Su et al.^[19]^ experienced better exposure of the ascending aorta during the proximal anastomosis. Another authors advocated operating on the right side while feeling comfortable performing such operations^[20]^. Bonde & Campalani^[8]^, Abdullah & Mazalan^[10]^ and Chakravarthy et al.^[13]^, even with different positions, experienced difficulties during exposure and anastomosis of the PDA with some hemodynamic instability, which obligated the first to convert the case to on-pump procedure.

Although not using CPB, we performed a triple coronary artery bypass surgery in a patient with dextrocardia and *situs inversus totalis*, with the surgeon standing on the left side of the patient being the unique change in the technical arrangement for the procedure that occurred without any complication. We chose to use the RIMA once the left one would not reach properly the LAD artery and would cross over the mediastinum. We have found that using deep pericardial traction sutures with minimal inotropic support allowed to us to obtain good exposure of the lateral and posterior walls of the heart without inducing hemodynamic instability. The postoperative course was uneventful, the patient was discharged home eight days later and one year after the operation he remained in good clinical condition and no further complication. A CT coronary angiography confirmed the viability of all grafts 9 months after the surgery.

**Table:** 

Authors' roles & responsibilities	
CJTK	Conception and design study; realization of operations and/or trials; analysis and/or data interpretation; manuscript redaction or critical review of its content; final manuscript approval
FB	Manuscript redaction or critical review of its content; final manuscript approval
ANM	Realization of operations and/or trials; final manuscript approval
RTT	Realization of operations and/or trials; final manuscript approval
FGJ	Final manuscript approval
